# Cellular Therapies in Systemic Sclerosis: Recent Progress

**DOI:** 10.1007/s11926-015-0555-7

**Published:** 2016-03-04

**Authors:** Femke C. C. van Rhijn-Brouwer, Hendrik Gremmels, Joost O. Fledderus, Timothy R. D. Radstake, Marianne C. Verhaar, Jacob M. van Laar

**Affiliations:** Department of Nephrology and Hypertension, Division of Internal Medicine and Dermatology, University Medical Center Utrecht, P.O. Box 85500, 3508 GA Utrecht, The Netherlands; Department of Rheumatology & Clinical Immunology, Division of Internal Medicine and Dermatology, University Medical Center Utrecht, P.O. Box 85500, 3508 GA Utrecht, The Netherlands

**Keywords:** Systemic sclerosis, Cellular therapy, Hematopoietic stem cell transplantation, Mesenchymal stromal cells, Dendritic cells, Regulatory T cells

## Abstract

Systemic sclerosis (SSc) is a rare autoimmune connective tissue disease with a high mortality and morbidity. While progress has been made in terms of identifying high-risk patients and implementing new treatment strategies, therapeutic options remain limited. In the past few decades, various cellular therapies have emerged, which have been studied in SSc and other conditions. Here, we provide a comprehensive review of currently available cellular therapies and critically assess their merit as disease-modifying treatment for SSc. Currently, hematopoietic stem cell transplantation is the only cellular therapy that has demonstrated clinical effects on the immune system, neoangiogenesis, and fibrosis. Robust mechanistic studies as well as clinical trials are essential to move the field forward.

## Introduction

Systemic sclerosis (SSc) is an autoimmune disease characterized by inflammation, skin fibrosis, and vasculopathy. Key clinical manifestations are skin tightening and Raynaud’s phenomenon. Gastro-intestinal, vascular and cardio-pulmonary complications may develop as disease progresses; the latter are the most common cause of death in SSc [1, 2]. Based on the extent of skin involvement, systemic sclerosis can further be classified as limited cutaneous systemic sclerosis (lcSSc) and diffuse cutaneous systemic sclerosis (dcSSc) [3].

Despite considerable advances in the treatment of autoimmune diseases, SSc remains a disease with high mortality and morbidity. The cumulative 5-year mortality for SSc patients from diagnosis is 25 %, with better outcomes for patients with lcSSc and worse outcomes for patients with dcSSc [4]. Currently available therapies are targeted to treating organ manifestations and while it has been shown that intravenous cyclophosphamide can halt progression of pulmonary and cardiac fibrosis, a true cure is lacking [5•, 6]. New therapeutics which have demonstrated spectacular results in other autoimmune diseases confer modest benefits at most in SSc, if effective at all [7]. Therefore, continuing the search for therapeutic options for SSc patients is essential.

SSc involves dysfunctional processes on several levels: the immune system, the vascular system, and extracellular matrix production [8–10]. A therapy which acts on all three levels can be considered a true disease-modifying therapy for SSc. Cellular therapies may be able to fulfill this potential. In this review, we provide an overview of currently available cellular therapies and critically review the available literature to evaluate the potential of cellular therapies in the treatment of SSc.

## Cellular Therapy: General Considerations

Cellular therapy can be defined as the administration of cells to achieve a therapeutic effect. This was first conceived in the late nineteenth century, starting with the implantation of xenogenic parathyroid tissue to treat hypoparathyroidism after thyroidectomy. The initial successes reported led to attempts to treat conditions ranging from Down’s syndrome to cancer with cells or extracts of animal or fetal origin. However, its efficacy remained uncertain and ultimately, as reports about zoonoses and allergic reactions emerged, interest in cellular therapy declined [11–13].

The advent of chemotherapeutics and the development of hematopoietic stem cell therapy (HSCT) rekindled the development of cellular therapies, as it could be established that administration of cells led to a demonstrable therapeutic effect. Studies focusing on bone marrow components identified cell populations possibly responsible for the restoration of hematopoiesis, leading to preclinical and clinical studies examining the effect of transplantation of these cell types. Insights into the role of the immune system in malignancy led to the development of cellular strategies to harness immune cells against malignant cells.

Nowadays, cellular therapies range from stem cell transplantation to highly selective cellular vaccines that can target specific immune cells. Other options include the transplantation of cells to restore tissue structure and function or to deliver specific cytokines and other paracrine factors to sites of inflammation. Cellular therapies are utilized in a wide variety of clinical settings, including autoimmune disease [14–18]. Table 1 lists the therapies currently available for autoimmune disease.

**Table 1:** Summary of cellular therapies in autoimmune disease

	Effects on immune system	Neoangiogenesis	Anti-fibrotic effects	Level of evidence
HSCT	+++	++	+++	Phase III clinical trial in SSc
MSC	++	+++	+	Case reports, case series in SSc
MNC	Unknown	+++	++	Phase 1 clinical trials in SSc
ADC	Unknown	++	++	Phase 1 clinical trials in SSc
DC	++	Unknown	Unknown	Phase I clinical trial in AD
Tregs	++	In vitro studies: possible role in angiogenesis	In vitro studies: inhibit secretion of pro-fibrotic factors	Phase 1 clinical trials in AD

The columns represent the desired qualities of a cellular therapy for SSc; the number of + depicts in what extent the cellular therapy possesses a quality. Level of evidence refers to available clinical evidence in AD

*AD* autoimmune disease, *HSCT* hematopoietic stem cell transplantation, *DC* dendritic cells, *Tregs* regulatory T cells, *MNC* mononuclear cells, *MSC* mesenchymal stem cells, *ADC* adipose tissue-derived cells

Cellular therapies originated from different clinical needs, reflected in a diversity of treatment protocols. Essential steps in every therapy are selection and isolation of the desired cell type, ex vivo manipulation of the cells, and finally administration of the cells to the patient.

### Cell Types and Isolation

**Fig. 1:**
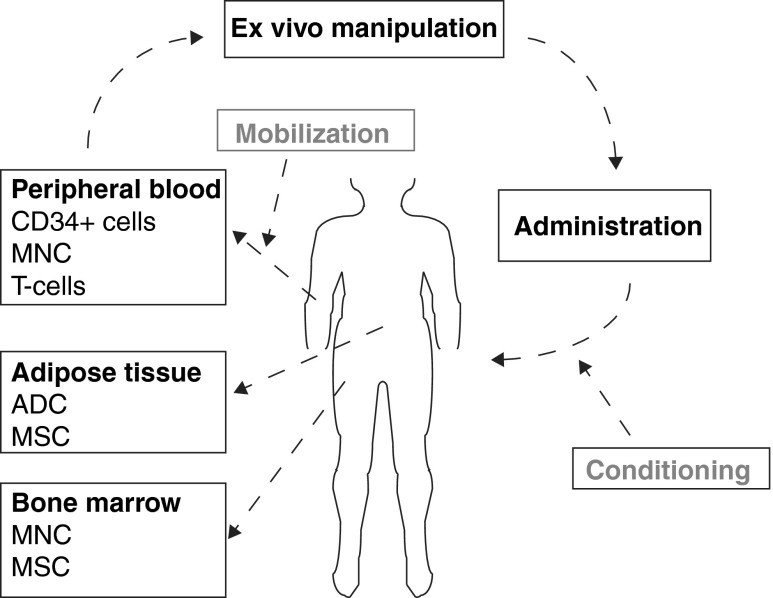
The process of cellular therapy. Cells can be isolated from various sources. Subsequent ex vivo manipulation allows isolation of specific cell types, differentiation of cells into the desired cell type, or prolonged culturing to expand cell numbers. Cells can then be administered. Preparation of the patient or donor may be necessary prior to isolation (mobilization) or administration (conditioning)

Cell types used in cellular therapy can be either autologous or allogeneic and can be harvested from various tissues (Fig. 1). Methods to obtain source material for cellular therapy range from venipuncture to the harvesting of specific tissues under regional or general anesthesia. More complex regimens include the pharmacological mobilization and subsequent apheresis of cells.

### Ex Vivo Manipulation

The desired cell types are isolated from the source material with specialized techniques such as magnetic bead separation, culturing on selective media or fluorescence-assisted cell sorting. Some approaches require further manipulation. For instance, scarcity of a cell type in vivo may necessitate ex vivo propagation, which can pose cell-specific challenges. Another approach is to differentiate an abundant cell type (e.g., monocytes) into the desired cell type using specific stimuli.

An important consideration is that protocols involving administration of cells to humans should comply with Good Manufacturing Practices (GMP) and local regulations for quality assurance and manufacturing requirements.

### Administration

Depending on the application, cells can be administered intravenously, intra-arterially, intramuscularly, or subcutaneously. Some cellular therapies require the use of adjuvant medication to induce immune suppression or immune ablation prior to administration. Administration protocols are often based on local experience and are rarely standardized, with HSCT as a notable exception.

## Hematopoietic Stem Cell Transplantation

HSCT was the first cellular therapy to be applied in refractory autoimmune disease. Originally developed in hemato-oncology, HSCT is used to reconstitute the hematopoietic niche after chemotherapy treatment or irradiation to obliterate malignant cells. HSCT consists of a mobilization phase to harvest CD34+ hematopoietic stem cells followed by administration of lympho-ablative chemo(radio)therapy to ablate the immune system (‘conditioning’) and subsequent reinfusion of the autologous graft.

### Rationale

Administration of high doses of cyclophosphamide and anti-thymocyte globulin leads to ablation of the immune system, including self-reactive immune cells. Subsequent administration of CD34+ hematopoietic stem cells regenerates a functional self-tolerant population [19]. After HSCT, less pro-inflammatory cytokines are produced and serum levels of anti-Scl-70, an antibody highly specific to SSc, gradually drop. Not all cell populations are completely “replaced” [20]. This suggests that a complete reconstitution of the immune system is not necessary for a treatment effect.

In SSc patients, regression of skin fibrosis in skin biopsies was seen after HSCT [21], implying that processes involved in dermal fibrosis can be reversible. Levels of pro-fibrotic cytokines were demonstrated to be lower after HSCT [20]. However, the effect on cardiac and pulmonary fibrosis seems to be limited. Patients demonstrated an initial improvement in ground glass aspect on high-resolution computed tomography (HRCT), but a subsequent deterioration to pre-transplant status was found at 24 months and then remained stable until 60 months follow-up. Improvement in modified Rodnan skin score (mRSS) was sustained. Importantly, lung function test results remained stable [22].

Neoangiogenesis has been observed in skin biopsies and capillaroscopy of SSc patients treated with HSCT [23, 24]. This suggests a beneficial effect of systemic immune suppression on angiogenesis. Another contributing factor might be the administration of CD34+ cells themselves. Administration of bone marrow-derived CD34+ cells to patients with limb ischemia has been reported to promote neovascularization [25].

### Clinical Trials in SSc

Uncontrolled studies in SSc have demonstrated that HSCT can halt disease progression and reverse skin fibrosis and suggested it might even reduce mortality [26–28]. The next step to evaluate HSCT for SSc was to compare its effectiveness and safety to intravenous cyclophosphamide pulses, the only treatment option for progressive SSc.

The American Scleroderma Stem Cell versus Immune Suppression Trial (ASSIST trial) was the first published, randomized trial to examine the possible superiority of HSCT as compared to six pulses of cyclophosphamide in SSc. Nineteen patients were included. Interim analyses were part of the trial design. This led to the early termination of the trial: HSCT led to a lasting remission, whereas patients treated with cyclophosphamide experienced disease progression after 1 year. No patients died during the trial [29].

The Autologous Stem cell Transplantation International Scleroderma (ASTIS) Trial was an international randomized multi-center phase III trial comparing HSCT to 12 successive monthly pulses of cyclophosphamide. Patients (*n* = 156) with early progressive systemic sclerosis were included in 29 European centers. HSCT was shown to have a higher treatment-related mortality in the first year (10.1 versus 0 % in the cyclophosphamide group) but showed a better survival overall when compared to the cyclophosphamide group. Furthermore, mRSS, pulmonary function, and quality of life significantly improved in the HSCT group as compared to the cyclophosphamide group, demonstrating that HSCT is a true disease-modifying treatment. Notably, seven of the eight patients who died due to treatment-related causes were smokers, indicating that such patient factors can play an important role in the success or failure of a systemic treatment. This trial also underlines the need to optimize HSCT protocols and patient selection, as the eight treatment-related deaths occurred during conditioning [30••].

The Scleroderma: Cyclophosphamide or Transplantation (SCOT) Trial, conducted in North America, included 75 systemic sclerosis patients with severe involvement of skin and internal organs to compare HSCT to cyclophosphamide pulses, albeit with lower doses for conditioning than the ASTIS Trial. This trial added total body irradiation to the conditioning regimen, with lung and kidney shielding to prevent radiation-induced organ complications [31]. Inclusion has been completed, data regarding the primary endpoint is expected in due course.

### Current Status and Future Perspectives

Beneficial effects of HSCT on immunomodulation, fibrosis, and angiogenesis have been demonstrated in SSc patients. This suggests that the immune system plays a key role in various processes including some that are not traditionally associated with the immune system.

The results of the ASTIS Trial established HSCT as a true disease-modifying treatment of SSc leading to inclusion of HSCT into clinical protocols for SSc patients. Further studies will have to focus on optimizing both the treatment as well as patient selection: how can we reduce treatment-related mortality while still maintaining strong SSc-related outcomes? A recently started study (Table 2) will examine the effect of a different, less intensive, conditioning regimen.

**Table 2:** Cell therapy trials in SSc registered on clinicaltrials.gov (search date: 4 September 2015)

Identifier	Type	Stage	Phase	Country
NCT00622895	Allogeneic HSCT, nonmyeloablative conditioning	Recruiting	Phases 1–2	USA
NCT01895244	HSCT	Recruiting	Phase 2	Germany
NCT02213705	MSC iv administration	Recruiting	Phases 1–2	France
NCT02206672	Facial implantation of autologous adipose tissue	Recruiting	Phases 1–2	France
NCT02396238	Autologous adipose tissue	Not yet started	Phase 2	Not provided
NCT00962923	Intravenous MSC	Unknown	Phases 1–2	China
NCT01413100	HSCT	Recruiting	Phase 2	USA
NCT00849745	HSCT less toxic conditioning	Recruiting	Phase 1	USA

## Mesenchymal Stem Cells

In 1974, Friedenstein et al. first discovered the presence of non-hematopoietic stem cells in the bone marrow [32]. These were later named mesenchymal stem or stromal cells (MSCs) for their potential to differentiate into various mesenchymal tissues [33]. MSCs are located on the outside of the bone marrow sinusoids, support circulation in the bone marrow, and play a role in hematopoiesis [34]. MSCs are isolated from bone marrow by plastic adherence—MSCs will adhere to a culture flask, whereas other cells can be washed away after 1 day. The low prevalence of MSCs in bone marrow necessitates further ex vivo expansion prior to administration.

MSCs are currently used therapeutically for graft-versus-host-disease and other applications are under investigation. MSCs have been safely administered to patients suffering from a wide variety of diseases, including autoimmune disease [35, 36••].

### Rationale

In vitro and vivo studies have demonstrated that MSCs can home to injured tissue and secrete factors that suppress inflammation and improve angiogenesis [37–39]. However, just administering the secretions of MSCs may not be sufficient as the MSCs’ secretome is influenced by local stimuli, e.g., inflammation or hypoxia [40]. Cell-to-cell interactions are also essential to immunomodulation and regenerative effects [41–43]. MSCs express low levels of MHC type II and can survive in an allogeneic host through immunomodulation, allowing allogeneic transplantation.

MSCs have demonstrated a potent immunosuppressive effect in steroid-resistant graft-versus-host disease (GVHD) [44]. Administration of allogeneic MSCs to SLE patients in an uncontrolled study was shown to be safe and effective, reportedly inducing remission in 50 % of patients after 4 years [45].

Anti-fibrotic effects of MSsC were seen in GVHD patients [46, 47], supporting the observation of a decrease in mRSS in SSc patients (below). As MSCs can secrete collagen and transforming growth factor beta (TGF-β), the anti-fibrotic effect is most likely due to the immunomodulatory effects of MSCs which may reduce production of pro-fibrotic factors by immune cells.

In animal models of ischemia, neovascularization after MSC administration has been demonstrated, leading to successful but small clinical trials that explored the use of MSCs in critical limb ischemia or chronic ulcers [48•]. Indications of formation of new vessels in a SSc patient were provided by angiography and histology [49]. MSCs secrete factors that stimulate neoangiogenesis and can activate local cells to migrate to sites of injury [37, 50].

### Clinical Trials in SSc

Clinical use of MSCs for SSc was described in two case reports and one case series (Table 3) [49, 51, 52]. Patients were suffering from extensive skin necrosis and/or pulmonary and cardiac fibrosis. All three reports noted substantial healing of ulcers and necrotic skin areas after intravenous administration of MSCs. Decrease of mRSS was also reported. Angiography findings indicated neovascularization. Leukocyte numbers did not change, though Christopeit et al. reported an increase of the relative fraction of CD137L-positive CD4+ and CD8+ cells after 14 days, suggesting that the effects of MSCs are mediated by CD137L+ T cells [51]. No adverse events related to MSC administration were reported. Thus, MSC administration, even allogeneic MSC, appears to be safe in SSc patients, but the small number and diversity of the patients treated do not allow conclusions regarding efficacy.

**Table 3:** Cellular therapy in SSc: published trials

Autologous stem cell transplantation in SSc						
	Year	*N*	Conditioning regimen	Comparator	Outcomes	Treatment-related mortality
ASSIST	2006–2009	19	Cyclophosphamide 200 mg/kg Rabbit ATG 7.5 mg/kg	6× iv cyclophosphamide 1000 mg/m^2^ (monthly)	Lasting decrease in mRSS Improved forced vital capacity	None
ASTIS	2001–2009	156	Cyclophosphamide 200 mg/kg Rabbit ATG 7.5 mg/kg	12× iv cyclophosphamide 750 mg/m^2^ (monthly)	Longer event/progression-free survival Decrease in mRSS Improvement of vital capacity	10.1 %
SCOT	2005--present	75	Cyclophosphamide 120 mg/kg 90 mg/kg equine ATG Total body irradiation (800 cGy)	12× iv cyclophosphamide (monthly) 1× 500 mg/m^2^, then 750 mg/m^2^	[n/a]	[n/a]
Other cellular therapies						
	Year	*N*	Administration route	Dose and cell type	Outcomes	Adverse events
Christopeit et al.	2008	1	iv	1 × 10^6^/kg body weight autologous BM-MSC	Healing of ulcers (from 17 to 0.4 cm^2^) mRSS decreased (−14)	None
Guiducci et al.	2010	1	iv	1 × 0.9 × 10^6^ and 2 × 0.8 × 10^6^ autologous BM-MSC	Substantial healing of necrosis, revascularization of lower limb	None
Keyszer et al.	2011	5	iv	1 × 10^6^/kg body weight allogeneic BM-MSC	Healing of skin ulcers, trend toward improvement of mRSS	None
Takagi et al.	2014	11	im + debridement and skin grafting	~ 5.42 × 10^7^ autologous BM-MNC	Ulcers in 10/11 patients completely healed. VAS improved in all patients.	No treatment-related complications
Nevskaya et al.	2009	2	im	11 × 10^6^ autologous BM CD34+ selected cells	Ulcer healing, reduction of Raynaud’s attacks	None reported
Kamata et al.	2007	3	im	BM: MNC: 7.7 × 10^8^, CD34+: 3.5 × 10^6^ PB: MNC: 3.5 × 10^8^, CD34+ 0.14 × 10^6^	Significant decrease in pain, increase in TcPO_2_ at 1 month	None reported
Ishigatsubo et al.	2010	8	im	~1,65 × 10^9^ autologous BM-MNC	Healing of ulcer in all patients, recurrence in 2 patients	No treatment-related adverse events
Granel et al.	2014	12	Injection along neurovascular bundles	3.76 ± 1.85 × 10^6^/finger autologous ADC	Decrease of Raynaud score and CHFS. Increase in grip strength	Paresthesia in 1 finger, pain in 1 finger, resolved after 2 weeks
Scuderi et al.	2013	6	sc	4 × 10^6^–8 × 10^6^ cultured autologous ASC in hyaluronic acid suspension	No local progression (all patients), regression of dyschromia (4 patients), skin softening (5 patients)	Small areas of ecchymosis
Del Papa et al.	2014	14	Injection at the base of the affected finger	Dose not provided, Autologous ADC	Healing of cardinal ulcer in all patients, mean 4.23 weeks. Reduction in VAS, no analgesic use after 1 month 60.7 % new capillaries in treated finger	None

Some trials also included patients with other conditions than SSc

*N* number of SSc patients in the trial, *iv* intravenous, *im* intramuscular, sc subcutaneous

### Current Status and Future Perspectives

Clinicaltrials.gov lists one trial examining intravenous administration of MSCs in SSc, its status is unknown (Table 2). As MSCs seem to have a favorable safety profile in severely affected SSc patients and given the successes reported, it is surprising that initial success of MSC administration has not yet led to clinical trials in SSc. Due to their status as “advanced therapy medicinal product,” GMP-grade MSCs are readily available in major medical centers in Europe.

Data from a recent meta-analysis on clinical studies where MSCs were administered intravenously do not indicate a higher risk of malignancy, ectopic transformation, or acute toxicity [36••] but it is important to remain vigilant as MSCs continue to be implicated in tumor transformation and metastasis. Furthermore, as a majority of infused MSCs become trapped in the lungs after systemic infusion [53, 54], more evidence as to the safety in SSc patients with lung involvement is required.

## Local Implantation of Cells

Promising results following local implantation of bone marrow-derived cells and adipose tissue-derived cells in critical limb ischemia motivated researchers to evaluate this as treatment for non-healing ulcers and hand and skin complaints in SSc.

### Bone Marrow-Derived Cells

The discovery of endothelial progenitor cells led to the therapeutic administration of progenitor cells to promote angiogenesis [55]. As endothelial progenitor cells are very rare in the blood but abundant in bone marrow, the effect of administration of CD34+ mononuclear cells (MNC) derived from bone marrow was investigated. Promising results following local implantation of bone marrow-derived cells and adipose tissue-derived cells in critical limb ischemia motivated researchers to evaluate this as treatment for non-healing ulcers and hand and skin complaints in SSc.

### Rationale

Intramuscular injection of bone marrow-derived cells creates depots of cells, reducing the need to home through a compromised vascular system and enabling cell–cell contact [56]. CD34+ cells were, like MSC, thought to home, engraft, and differentiate into new vascular structures.

### Clinical Trials

In four small uncontrolled trials, bone marrow or peripheral blood-derived MNC injections were studied as treatment for ulcers of the upper and lower extremity in SSc (Table 3). Dosages and reported injection technique varied, utilizing 20 to 70 injections. A decrease in visual analogue scale (VAS) for pain was noted in all studies, and some reported changes in objective studies of vascular function, e.g., transcutaneous oxygen pressure. Local implantation was well tolerated. Self-limiting local swelling or hematoma occurred in some patients but did not lead to local complications [57–60].

### Adipose Tissue-Derived Cells

Adipose tissue-derived cells (ADC) can be isolated from tissue obtained through liposuction. Cellular therapies based on ADC can involve either freshly isolated cells or expanded cells.

### Rationale

Few studies have been conducted utilizing freshly isolated autologous adipose tissue; most studies focus on ex vivo expanded MSC isolated from adipose tissues, of which the angiogenic potential has been well characterized in both preclinical and clinical studies [48•]. It can be hypothesized that pericytes or progenitor cells present in the vascular stroma are responsible for the clinical effect [61•]. The observed anti-fibrotic effects suggest that unexpanded ADC also have immunomodulatory properties.

### Clinical Trials

Three uncontrolled trials regarding the use of ADC in SSc have been performed (Table 3); two trials used unexpanded ADC and one trial used expanded cells. ADC administration reduced skin tightness, decreased Raynaud’s symptoms, and promoted the healing of digital ulcers. A decrease in VAS for pain was also seen. Del Papa et al. reported significant growth of capillaries in the treated finger. Adverse events were limited to local complaints after implantation, though the injection technique in the study by Granel et al. caused paresthesia in one patient and finger pain in another patient, which did resolve spontaneously [61•, 62, 63].

### Current Status and Future Perspectives

To further develop local cell implantation, key questions still need to be answered. First of all, comparative studies into cell types used are needed. Recent results from both preclinical and clinical studies in limb ischemia indicate that MSCs seem more effective at neovascularization than MNC to treat limb ischemia [64, 65]. However, results from studies with unexpanded ADC indicate that unexpanded cells may also be effective. Additionally, the optimal dose, administration route, number of injections, and volume to be injected need to be determined.

The studies summarized here provide some reassurance that the trauma of injection does not increase fibrosis and that injected cells do not differentiate inappropriately in a pro-fibrotic environment. However, additional data from controlled studies to establish the efficacy and safety of local cell implantation is needed.

## Tolerogenic Dendritic Cells

Dendritic cells (DCs) are key players in the immune system because of their essential role in both immune activation as antigen-presenting cells as well as their ability to induce tolerance. DCs maintain tolerance by recognizing self-reactive T cells; DCs can subsequently deactivate them or induce differentiation of regulatory T cells which in turn can suppress the self-reactive effector T cells.

DCs with a tolerogenic effect (tolDCs) can be generated ex vivo and then administered to induce tolerance. DCs are physiologically present in low numbers; therefore, for therapeutic purposes, monocytes differentiated to DCs are often used [66]. After differentiation, the generated DCs are exposed to specific compounds or genetically manipulated to induce tolerance. Tolerance to a specific antigen can also be achieved, but stimulation with self-antigens is not required to achieve a tolerogenic effect [67].

### Rationale

In SSc, a multitude of immune cells is involved. Harnessing the tolerogenic functions of DCs may allow manipulation of an out-of-control immune response, as is present in autoimmune disease [68]. A major advantage of this approach is that only self-reactive T cells are disabled, preventing general immunosuppression.

There is no direct evidence that the antigen presenting/tolerogenic functions of DCs are disturbed in SSc. Current thought suggests a pro-inflammatory phenotype of DCs of SSc patients. Monocyte-derived and myeloid dendritic cells in patients with SSc produce more cytokines in response to TLR stimulation [69]. As patients with SSc have an interferon 1 signature and DCs are implicated in interferon-1 production, it is suggested that this signature can be partly explained by aberrant secretion of interferon 1 by plasmacytoid DCs [70]. In SSc patients, levels of CXCL4, a chemokine secreted by plasmacytoid DCs, strongly correlate with disease activity and progression. In vitro and in vivo studies by Van Bon et al. strengthen the evidence for a possible role of DCs in the pathogenesis of SSC [71•]. DCs have been shown to be involved in angiogenesis [72]; it is unknown whether administration of tolDCs influences this aspect.

### Clinical Trials

Therapeutic DCs are widely studied in oncology, though their application is intended to achieve immunity against specific antigens, not tolerance; therefore, data from oncology trials might not be applicable to autoimmune disease [73].

The first clinical trial reporting the use of tolDCs for an autoimmune condition examined their effects in DM type 1. Ten patients received autologous DCs in four doses, doses were administered intradermally biweekly. Three patients received “control DCs” which had not been manipulated, and seven patients received tolDCs which had been manipulated ex vivo with antisense nucleotides to block expression of co-stimulatory molecules. No adverse events were detected. In the verum arm, a transient increase in B220+ CD11c- B cells was found, possibly caused by administration of the DCs. However, this study was designed to determine safety, not efficacy [74].

Another phase 1 study studied the safety and possible efficacy of administration of “Rheumavax” in patients with rheumatoid arthritis. Rheumavax consists of autologous monocyte-derived DCs which were exposed to a tolerance-inducing compound and subsequently exposed to four citrullinated peptide antigens. Adverse events were self-limiting, and distinct effects on regulatory T cell populations could be detected. Disease activity scores decreased in treated patients in comparison with historical controls [75].

### Current Status and Future Perspectives

tolDCs are not yet under development for SSc. Clinical trials in oncology have demonstrated that modified DCs can have off-target effects, contributing to auto-immunity. While there are several (auto)antibodies associated with SSc, it remains a question to what extent the blocking of antibody-specific cells will benefit SSc patients.

DCs have been implicated in fibrosis of various organs. In experimentally induced lung fibrosis, DCs were shown to accumulate with pathogenic T cells, whereas blocking of DC surface molecules was associated with less fibrosis, suggesting that antigen presentation by DCs to T cells plays a role in the pathogenesis of fibrosis [76]. Additionally, a paracrine role for DCs has not been excluded. If dendritic cells in SSc are pro-fibrotic, infusing them may exacerbate disease, similar to concerns regarding DC use in SLE [77].

## Regulatory T Cells

Regulatory T cells (Tregs) modulate the immune response by regulating effector T cells and maintain tolerance by recognizing self-reactive T cells and inhibiting them. Tregs can be identified by their surface expression of CD25, CD4, and FOXP3. As a therapy, Tregs can be infused to achieve general immune suppression, and they can also be modified or primed ex vivo to act on T cells specific to an antigen [78, 79]. Tregs have to be expanded prior to use.

### Rationale

In animal models of autoimmune disease, the administration of ex vivo expanded autologous Tregs has been shown to ameliorate disease. In clinical trials, variations in levels of immune cells before and after treatment have been noted, though its significance remains unknown as these trials were mainly designed to assess safety [80]. T cell activation in response to a yet unknown self-antigen plays an important role in SSc pathogenesis.

T cell infiltrates can be found in the skin of SSc patients, and a shift to the Th2-phenotype has been reported [81]. Experimentally induced SSc in animal models could be treated by blocking T cell cytokines like IL-6. The administration of Tregs may therefore reduce self-reactive T cell activity.

Abnormalities have been found in Tregs of SSc patients as well. SSc patients have more circulating Tregs and less skin Tregs than healthy controls [82]. Circulating SSc Tregs were found to display fewer CD62L, and CD69 surface receptors SSc Tregs also secrete less Treg specific cytokines. The levels of CD69 and TGF-β were found to correlate with the suppressive action on CD4+ T cells. Radstake et al. further show that the suppression of Treg function in SSc patients is mediated by a yet unknown plasma factor: upon addition of serum of SSc patients to healthy Tregs, suppression of CD4+ cells was reduced [83]. There are indications that only skin Tregs, not circulating Tregs, secrete effector T cell cytokines [84].

Tregs may play a role in fibrogenesis by controlling other immune cells involved. In an animal model of lung fibrosis, early depletion of Tregs led to favorable outcomes whereas late depletion of Tregs led to increased fibrosis [85]. A recent animal study in liver injury showed that Tregs reduce fibrosis in fibrogenic settings by inhibiting CD8+ and Th17+ cells [86].

FOXP3+ Tregs appear to be essential for angiogenesis in the lung. Induction of lung ischemia in mice led to an increase in FOXP3+ Tregs in the lung. Depletion of Tregs severely impaired angiogenesis. Further analysis showed that Treg-deficient mice had less macrophages in their ischemic lungs, suggesting that they are required for the attraction of macrophages which aid angiogenesis [87]. As Treg numbers in SSc skin are low, administration of Tregs can possibly aid angiogenesis.

### Clinical Trials

Treg administration was first studied in GVHD, as a low number of Tregs correlates with a higher risk of GVHD after allogeneic SCT. Trzonkowski et al. intravenously administered autologous Tregs to two patients with refractory GVHD. One patient (chronic GVHD) responded well, immunosuppressives could be stopped, and lung function improved. The second patient had rapidly progressing acute GVHD and, although Treg infusion alleviated symptoms (comparable with immunosuppressives), the patient eventually died, possibly due to the fact that Tregs were infused in a relatively late stage of the disease [88]. Brunstein et al. administered umbilical cord-derived allogeneic Tregs prior to umbilical cord blood transplantation in 23 GVHD patients. Infusion was well tolerated and no opportunistic infections occurred. As the primary goal of the study was to demonstrate safety, no definitive evidence for efficacy was gathered, but comparisons with historical controls were favorable [80]. Use of allogeneic Tregs hence appears to be safe.

Based on the promising results after Treg administration in GVHD patients, pilot studies in autoimmune disease were initiated. Marek et al. administered Tregs to 10 children diagnosed with diabetes mellitus type 1. No adverse events related to the treatment were observed. Compared to 10 matched controls, at 4 months posttreatment, treated patients required significantly less insulin per kilogram body weight, though glucose levels and HbA1c levels did not differ. Two doses were tested but no differences between patients were seen. Interestingly, the administered dose was dependent on the amount of cultured Tregs, which suggests a possible relationship between circulating Tregs and the effect of transfusion. Desreumaux et al. treated 20 patients with refractory Crohn’s disease with ovalbumin specific Tregs. Three treatment-related reactions were observed and sensitivity to drosophila proteins in two patients, which emphasizes the importance of animal-free culturing conditions. Eight patients responded with an improvement in Crohn’s disease activity index (CDAI), though only three patients met the criteria for “disease in remission” (CDAI <150) [89].

### Current Status and Future Perspectives

Though there is a wealth of animal studies and a few clinical studies available to support the possible beneficial effect of Treg administration in autoimmune disease, it remains unknown if Treg administration can be truly useful in SSc. The suppressive effect of serum factors on Treg effector function of even healthy cells may negate the effect of administering extra Treg.

Furthermore, the question remains whether administration of Tregs can also ameliorate fibrosis in SSc patients. The results in the chronic GVHD patient reported by Trzonkowski are somewhat reassuring in this aspect; even long-lasting symptoms were alleviated.

## Conclusions

The past decade saw the establishment of HSCT as an effective treatment option for patients with rapidly progressive SSc without irreversible organ damage, leading to inclusion in clinical guidelines and treatment protocols. HSCT remains the only cellular therapy of which efficacy in SSc is indisputable, though at a price of considerable treatment-related mortality.

Systemic administration of MSC may be an attractive option for SSc patients ineligible for HSCT given the promising results reported in patients with extensive disease; however, safety and efficacy have yet to be established as clinical evidence consists of case reports, not formal clinical trials. Equally, patients suffering from digital ulcers and local skin complaints may benefit from local cell implantation, but, here too, solid clinical evidence is lacking.

tolDC and Treg administration hold the promise of modulating the immune system without immune-ablative therapy. The safety profile of DCs as determined from the pilot studies in AD is encouraging. However, further preclinical studies are needed to determine if this therapy can be translated to SSc, given the possible pro-fibrotic phenotype of DCs in SSc. Similarly, Treg administration appears to be safe but the discovery of a serum factor that negates the immunosuppressive functions of Tregs may mean that Treg-mediated immunosuppression cannot be achieved in SSc. This issue has to be addressed before clinical trials can be initiated.

### Future Directions

Even though cellular therapies are already being clinically evaluated in SSc and other conditions, questions regarding critical issues remain: cell type, dosage, optimal routes of administration, and long-term safety. These questions can only be addressed through additional preclinical and phase I studies.

Large scale, blinded, and controlled trials are then essential to validate earlier results, not only because of the heterogeneous course of disease and tendency of spontaneous improvement in SSc [90] but the nature of stem cell treatment carries a risk of a significant placebo effect. In the field of cardiology, where the effect of bone marrow administration on left ventricle function was studied in patients with a myocardial infarction, none of the large randomized placebo controlled trials (2009–2015) met their primary endpoint, whereas 63 % of the earlier less rigorously designed, uncontrolled trials (2002–2008) had demonstrated beneficial effects [91••]. Similarly, administration of bone marrow MNC was a promising treatment strategy in critical limb ischemia, but recently, a large, placebo-controlled trial failed to demonstrate a difference in outcomes between placebo and cell treatment [92]. This underlines the need for well-designed trials to provide the necessary evidence of safety and efficacy. Given the low prevalence of SSc and the complexity of cellular therapies including the regulations, (inter)national collaboration is essential to move the field forward.

A next step toward incorporation in clinical protocols is to determine the place of cellular therapy in the treatment regimen. Currently, cellular therapy is often viewed as a last resort, but ideally, treatment starts before irreversible damage has occurred. Levels of circulating cytokines and immune cells may provide a clue as to the optimal timing of cellular therapy. Treatment strategy trials are vital to help support treatment decisions. Identification of prognostic factors and determinants of successful treatment through careful analysis of clinical trial data will enable selection of patients most likely to benefit from the treatment.

Finally, as trials move from phase 1 to phase 3, gaining knowledge about synergistic or antagonistic interactions with current therapy becomes more important. Other options to be explored are combinations of cellular therapies, for instance, co-administration of multiple cell types.

It remains essential to critically assess each potential therapy strategy for its possible merit in SSc patients to avoid blindly following the latest hype in the cellular therapy. The continuing unmet clinical need of many SSc patients justifies ongoing fundamental and clinical research of candidate therapies.
